# Polyphenols: Natural Food-Grade Biomolecules for the Treatment of Nervous System Diseases from a Multi-Target Perspective

**DOI:** 10.3390/ph17060775

**Published:** 2024-06-13

**Authors:** Xinchen Wu, Yang Zhou, Yujiang Xi, Haimei Zhou, Zhengxiu Tang, Lei Xiong, Dongdong Qin

**Affiliations:** 1 The First School of Clinical Medicine, Yunnan University of Chinese Medicine, Kunming 650500, China; 15270868330@163.com (X.W.); zhouyang_113@163.com (Y.Z.); 18635337136@163.com (Y.X.); 2 School of Basic Medical Science, Yunnan University of Chinese Medicine, Kunming 650500, China; 17773572364@163.com (H.Z.); 18087067564@163.com (Z.T.); 3 Key Laboratory of Traditional Chinese Medicine for Prevention and Treatment of Neuropsychiatric Diseases, Yunnan University of Chinese Medicine, Kunming 650500, China

**Keywords:** polyphenols, nervous system diseases, autism-spectrum disorders, anxiety disorders, depression, sleep disorders

## Abstract

Polyphenols are the most prevalent naturally occurring phytochemicals in the human diet and range in complexity from simple molecules to high-molecular-weight polymers. They have a broad range of chemical structures and are generally categorized as “neuroprotective”, “anti-inflammatory”, and “antioxidant” given their main function of halting disease onset and promoting health. Research has shown that some polyphenols and their metabolites can penetrate the blood–brain barrier and hence increase neuroprotective signaling and neurohormonal effects to provide anti-inflammatory and antioxidant effects. Therefore, multi-targeted modulation of polyphenols may prevent the progression of neuropsychiatric disorders and provide a new practical therapeutic strategy for difficult-to-treat neuropsychiatric disorders. Therefore, multi-target modulation of polyphenols has the potential to prevent the progression of neuropsychiatric disorders and provide a new practical therapeutic strategy for such nervous system diseases. Herein, we review the therapeutic benefits of polyphenols on autism-spectrum disorders, anxiety disorders, depression, and sleep disorders, along with in vitro and ex vivo experimental and clinical trials. Although their methods of action are still under investigation, polyphenols are still seldom employed directly as therapeutic agents for nervous system disorders. Comprehensive mechanistic investigations and large-scale multicenter randomized controlled trials are required to properly evaluate the safety, effectiveness, and side effects of polyphenols.

## 1. Introduction

More than one-third of the global population suffers from nervous system diseases, which are a major source of illness, sickness, and impairment. They also have one of the greatest prevalence rates linked to a higher burden of disease, which can have detrimental effects on family members [1,2]. Neurodevelopmental disorders, such as autism-spectrum disorders (ASD); neurodegenerative disorders, such as Alzheimer’s disease and Parkinson’s disease; mood disorders, such as anxiety, depression, and sleep disorders; psychiatric disorders such as different forms of schizophrenia; and other neurological disorders, such as aphasia and attention deficit hyperactivity disorder are commonly included in this category [3]. Nervous system diseases are a group of complex diseases involving the central nervous system. In addition to affecting the physiological functions of the body, abnormalities in the nervous system make the brain more susceptible to stimulation by the external environment, and this susceptibility, coupled with the stresses of life (e.g., difficulties encountered in the family or at work), greatly contributes to the onset of psychiatric disorders, with far-reaching impacts on the patient’s mental and psychological health [4,5,6,7].

The pathogenesis of nervous system diseases is complex and involves a variety of factors. Genetic factors play an important role in the pathogenesis of many neurological disorders [8]. Nervous system diseases early in life or congenital neurodevelopmental disorders cause the brain to respond differently to external stimuli, and stress causes abnormalities in neurons and affects the salient functions and neurodevelopment of the brain, which further affects their ability to interact and function with others [9,10,11,12].

Autism spectrum disorder is a complex neurodevelopmental disorder that often results in abnormal neurodevelopment and disruption of neuroplasticity in the brain early in an individual’s life; the etiology of the disorder is often based on genetic factors and environmental stimuli interacting with each other to cause structural and functional brain abnormalities [13,14,15]. The pathogenesis is related to oxidative stress, neuropeptides, nutrients, and immune abnormalities [16,17,18]. Research has revealed that approximately two-thirds of children with ASD experience chronic insomnia, sleep disorders affect about 80% of preschool-aged children with autism, and sleep issues are twice as common in children with autism as they are in children with typical development and children with other developmental conditions [19,20,21,22]. People with autism are more likely to experience sleep issues than those in the general population, because they have a genetic mutation that affects melatonin levels, a natural hormone that regulates sleep. Only 15% of their sleep time is spent in the rapid eye movement (REM) stage of sleep, which is essential for learning and memory retention [23]. Sleep disorders also influence the brain’s production of neurotransmitters, including dopamine and serotonin (5-hydroxytryptamine, or 5-HT), as well as other irregularities. These effects cause neuroinflammation and oxidative stress, which in turn predispose the body to mental illnesses such as sadness and anxiety at a later stage [24,25,26]. For nervous system diseases, the main treatments currently available include medications such as small molecule neurotrophins, immunosuppressants, anti-inflammatory and anti-infectious agents, lifestyle interventions, psychotherapy, surgery, and herbal or aromatherapy [27,28,29]. The use of natural remedies like polyphenols to treat nervous system diseases has gained popularity as a study area in recent years [30,31].

As organic substances derived from plants, polyphenols comprise at least one aromatic ring or benzene ring linking multiple hydroxyl functional groups [32]. The wide structural diversity of phenolic compounds includes more than 8000 compounds described in nature, which are usually categorized into two main groups based on the number of aromatic rings and the way they interact with each other, i.e., flavonoids (60%) and non-flavonoids [33]. Anthocyanins, flavan-3-oils, flavonoids, flavanones, and other subclasses of flavonoids are the several categories into which flavonoids fall. Flavonoids such as quercetin (QUE) are commonly found in the human diet and they usually reside as glycosides. Numerous studies have found thousands of these polyphenolic compounds in foods and plants, and they have been shown to have bioactivities (such as antibacterial and neuroprotective qualities) and antioxidant effects that may lower the risk of illness [34]. Phenolic acids (30%) and other polyphenols, such as lignans and stilbenes, are examples of non-flavonoids [35,36]. Phenolic acids are often found in conjugated form in bran and husk, and they can also be found in free form in fruits and vegetables. Stilbenes and lignans, which are present in grains like sesame seeds, are additional polyphenols, also found in wine and red wine [37,38]. Additionally, polyphenols are a potent antioxidant that is useful in the management of disorders linked to neuroinflammatory processes and oxidative stress. Reactive oxygen species (ROS) levels can be decreased and eliminated by supplementing with antioxidant vitamins and enzymes, such as vitamins C and E, carotenoids, and other antioxidants, to protect organisms from environmental stressors [30]. The adverse effects of conventional drugs such as calcitonin-norepinephrine reuptake inhibitors (SNRIs) and selective 5-hydroxytryptamine reuptake inhibitors (SSRIs), used to treat disorders of the nervous system, have been popularized more recently. Natural remedies high in polyphenols can be present in our regular diets and provide less negative effects than other pharmaceuticals. Polyphenols are likely found in a wide variety of everyday plant foods including fruits, vegetables, tea, and chocolate. Polyphenols are also frequently included in herbal remedies [39].

Previously, studies have evaluated the potential preventative and therapeutic benefits of polyphenols in nervous system diseases [40,41,42]. Despite a fair amount of research, the fundamental processes by which polyphenols function in disorders of the nervous system remain unknown. This paper reviews the effects of polyphenols on nervous system diseases such as autism, sleep disorders, anxiety disorders, and depression, including ex vivo and clinical studies. We have also reviewed mechanisms via which polyphenols have neuroprotective benefits.

## 2. Literature Searching Method

A thorough literature search for English-language papers was performed from January 2014 to May 2023. Current and relevant data were extracted from Internet sources such as Google Scholar, PubMed, SpringerLink, and Web of Science. The following key phrases were used alone or in combination: “polyphenols”, “phenolics”, “phenolic acids”, “stilbenes”, “lignans”, “flavonoids”, “biomolecules”, “dietary sources”, “nervous system diseases”, “autism-spectrum disorders”, “anxiety disorders”, “depression”, “sleep disorders”, “neuroprotective”, “anti-inflammatory”, “antioxidant”. The titles and abstracts were scanned to exclude any unrelated studies. A total of 143 studies concentrating solely on nervous system disease effects of polyphenols on health were searched, and articles having relevant data were examined. Review articles and original research based on animal studies and clinical trials were given top priority.

## 3. Role and Mechanisms of Polyphenols in the Treatment of Nervous System Diseases

**Figure 1:**
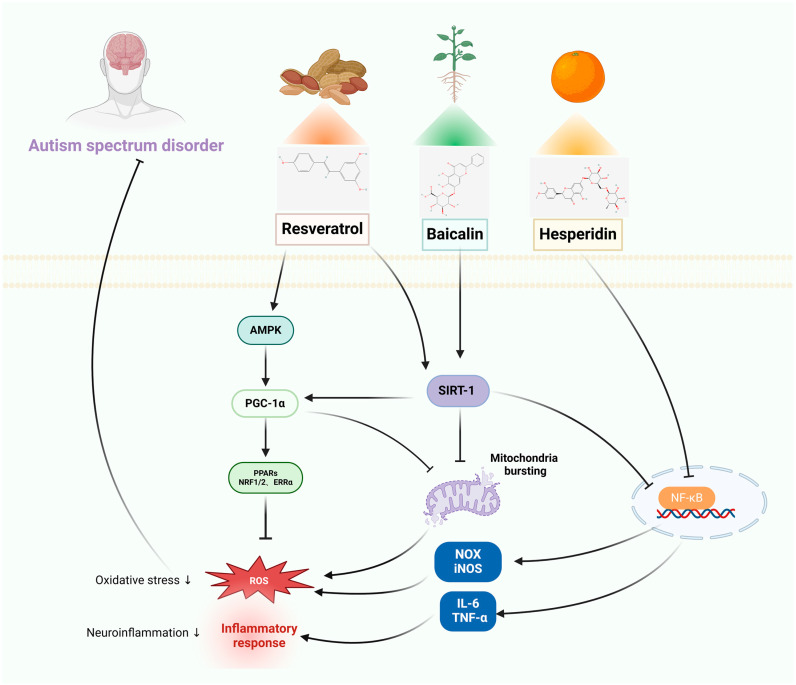
The therapeutic mechanism of polyphenols for ASD. ASD is caused by decreased levels of SIRT-1. Decreased SIRT-1 expression may increase NF-κB-mediated neuroinflammation and mitochondrial bursting. PGC-1α is a major regulator of mitochondrial bursting, ultimately elevating the levels of ROS, leading to oxidative stress and neuroinflammation, resulting in the development of ASD. Baicalin stimulates SIRT1/PGC-1α, decreasing mitochondrial bursting and the levels of ROS. Resveratrol activates AMPK and SIRT-1, which decreases the levels of ROS and neuroinflammation. Hesperidin inhibits oxidative stress and neuroinflammation via the NF-κB pathway.

**Figure 2:**
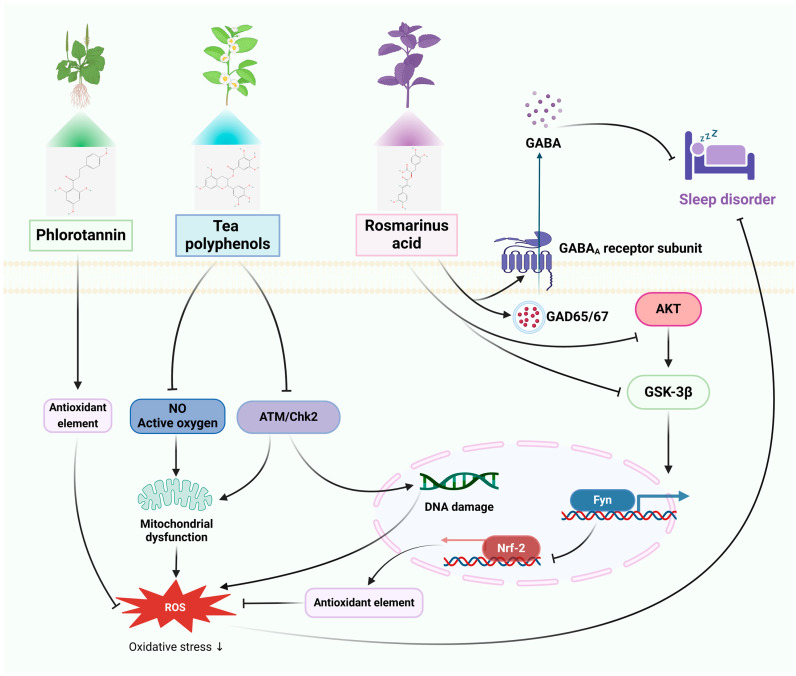
The therapeutic mechanism of polyphenols for sleep disorders. Sleep disorders are caused by chronic stress and impaired function of neurotransmitters. Phlorotannin inhibits the production of ROS. Tea polyphenols attenuate the production of nitric oxide (NO) and active oxygen to alleviate mitochondrial dysfunction, as well as enhance the expression and antioxidant capacity of p-ATM and p-Chk2 to reduce DNA damage. Rosmarinus acid exerts antioxidant effects by improving the Akt/GSK-3β/Fyn pathway to upregulate Nrf2 activity, and also increases the expression of glutamic acid decarboxylase (GAD65/67) and GABAA receptor subunits to improve sleep disorder.

**Figure 3:**
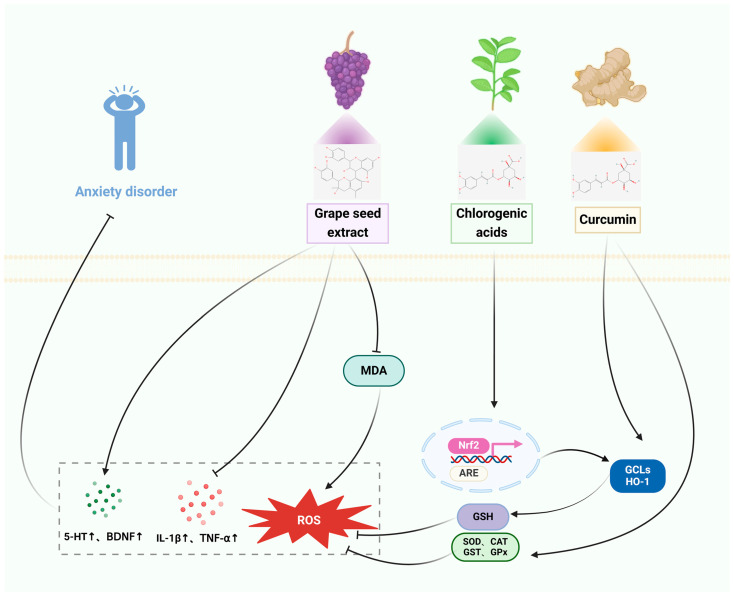
The therapeutic mechanism of polyphenols for anxiety disorder. Anxiety disorder is caused by extrinsic stress, with decreased Nrf2 expression leading to reduced antioxidant capacity and elevated ROS. Meanwhile, elevated CORT causes neuroinflammation and dysfunctional monoamine neurotransmitter secretion in the brain. Grape seed extract inhibits the release of MDA, ROS, IL-1β, and TNF-α, and promotes the secretion of 5-HT and BDNF. Curcumin enhances the activity of antioxidant enzymes to decrease the levels of ROS. Chlorogenic acids activate the Nrf2 signaling pathway to decrease the levels of ROS.

**Figure 4:**
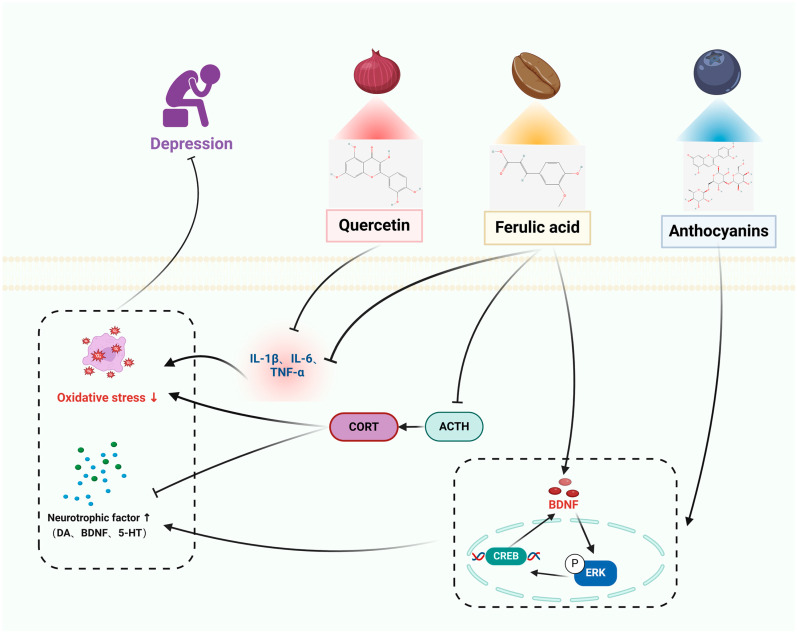
The therapeutic mechanism of polyphenols for depression. Depression is caused by extrinsic stress and dysregulation of neurotrophic factors in the brain. Quercetin and ferulic acid decrease the expression of the pro-inflammatory cytokines, including IL-1β, IL-6, and TNF-α. Anthocyanins and ferulic acid upregulate the release of BDNF through the ERK/CREB/BDNF signaling pathway. Ferulic acid decreases the levels of adrenocorticotropic hormone (ACTH) and corticosterone (CORT).

The pathophysiology of disorders of the nervous system is illustrated in Figure 1, Figure 2, Figure 3 and Figure 4, along with the main targets and possible methods of polyphenols in the treatment of nervous system diseases, including ASD, sleep disorders, anxiety disorders, and depression. Table 1 and Table 2 further illustrate the types of polyphenols, as well as the effects and mechanisms of in vitro and in vivo experiments and clinical trials.

### 3.1. Autism-Spectrum Disorders

Autism-spectrum disorders are neurodevelopmental illnesses characterized by very repetitive and/or restricted behaviors as well as trouble connecting and communicating with others [90,91]. The emergence of ASD has been linked to several variables, including oxidative stress, inflammation, immunological dysfunction, mitochondrial malfunction, and environmental toxicity [17]. A portion of the pathophysiology of ASD has been linked to neuroinflammation, which is typified by persistent glial responses that are unique to the central nervous system. Damage to brain tissue can result from neuroinflammation, which also causes aberrant neuronal development, an increase in pro-inflammatory cytokine production, and plaque formation [92]. A further element implicated in the etiology of ASD is oxidative stress, which is defined by cellular damage caused by reactive oxygen species, as demonstrated by increased lipid peroxidation, elevated reactive oxygen species, and other markers of oxidative stress. Additionally, it is believed that children diagnosed with ASD are more vulnerable to oxidative stress because of lower glutathione reserve capacity and unbalanced intracellular and extracellular glutathione levels [93,94]. Furthermore, cumulative damage and oxidative stress may affect the onset or severity of autism and its comorbidities by reducing the ability to produce functional mitochondria. Increased ROS production brought on by dysfunctional mitochondria may also cause chronic oxidative stress [95,96].

#### 3.1.1. In Vitro Studies

Mitochondrial biogenesis is initiated by an energy imbalance sensed by two pathways: the AMPK and the silent information regulator-1 (SIRT-1) [97]. Furthermore, SIRT-1 is a crucial modulator of several cellular functions [98]. SIRT-1 expression in the brain is significantly decreased in the hippocampus, prefrontal cortex, amygdala, and other regions. This further increases ROS production and oxidative stress, which in turn increases innate and adaptive immunological responses and exacerbates symptoms of autism [99]. It has been shown that upregulation of SIRT-1 protein expression correlates with neuroprotective effects in ASD. Resveratrol (RES) is a natural plant antitoxin for the treatment of neurological disorders associated with mitochondrial dysfunction, with neuroprotective, antioxidant, and anti-inflammatory properties [100,101]. Moreover, RES increases mitochondrial biogenesis and improves mitochondrial number by activating the SIRT1/PGC-1α signaling pathway in KGN cells [43], and further promotes mitochondrial biogenesis through activation of AMPK to regulate SIRT-1 protein activity in PCC12 cells [45]. The SIRT-1 signaling pathway plays a key role in mitochondrial biogenesis and is regarded as an endogenous neuroprotective mechanism. Down-regulation of SIRT-1 decreases PGC-1α expression and mitochondrial biogenesis, increases complex I dysfunction, increases oxidative protein levels, enhances the expression of activated caspase-3, and promotes hippocampal neuronal cellular injury [102]. Baicalin (BAI) is a flavonoid with multiple pharmacological activities [103]. This study discovered that BAI enhanced motor development, repetitive behaviors, and social impairments in rats prenatally exposed to valproic acid (VPA) modeling, which was performed on gestation day 12.5. Via downregulating caspase-3 expression and upregulating SIRT1 in SH-SY5Y cells, BAI prevents neuroinflammation and apoptosis [47]. It has been shown that activated B cells’ nuclear factor κ-light chain enhancer (NF-κB) controls the production of pro-inflammatory cytokines and the response to external stress [104]. In both animal models and the cerebellum and cortex of ASD patients, there is a significant alteration in the expression of NF-κB and its phosphorylation at the Ser536 site [105]. Aqueous fruit extract (AFE) of the date palm has many medicinal properties when consumed alone or in combination with other herbs [106]. It was revealed that AFE inhibits the NF-κB and MAPK signaling pathways in RAW264.7 macrophages, which results in anti-inflammatory actions [49]. Hesperidin (HES) has an outstanding neuroprotective effect and a large therapeutic potential in nervous system diseases [107]. By inhibiting oxidative stress, neuroinflammation, apoptosis, and cognitive consolidation, HES could reduce oxidative stress in BV-2 cells [51].

#### 3.1.2. In Vivo Studies

In a study of VPA-induced autism in rats, RES blocked VPA-induced changes in neuron numbers in the mPFC by improving parameters related to E/I balance, preventing an increase in the total number of neurons in the deeper layers of the neuron population, and preventing extensive damage to SOM+ neurons (one class of excitatory hypothalamic neurons) [44]. Additionally, in BTBR T+tf/J mice, RES alleviated neuroimmune dysregulation by blocking pro-inflammatory mediators and TLRs/NF-κB transcription factor communication and decreased the expression levels of TLR2, TLR3, TLR4, NF-κB, iNOS, and COX-2 mRNA in brain regions [46]. BAI increased SIRT-1 levels, restored antioxidant defense mechanisms, enhanced neuronal mitochondrial function, enhanced levels of mitochondrial adenosine triphosphate (ATP) and mitofusin-2 expression, as well as the absence of neuronal histological alterations in the brain tissues of rats who developed ASD because of exposure to VPA [48]. AFE ameliorates VPA-induced autism-like symptoms in rats by attenuating oxidative stress, up-regulation of Nrf2 and HO-1, down-regulation of caspase-3 by SIRT-1 and LC3 expression, and expression of NFκB in cerebellum and hippocampus [50]. It was found that HES exerts neuroprotective effects on fluoride-induced rats via PPAR-γ receptors [108]. There is also evidence that HES protects the nervous system in the context of lipopolysaccharide-induced neuroinflammation (through modulation of Tlr4/NFκB signaling) and amyloid β-induced neurodegeneration (through modulation of Nrf2/Tlr4/NFκB signaling). In neurons, brain-derived neurotrophic factor (BDNF) upregulates peroxisome proliferator-activated receptor γ coactivator 1-α (PGC-1α), which regulates energy metabolism and mitochondrial function/biogenesis. Oxidative stress is a major negative regulator of PGC-1a expression/activity [52]. Palmitoyl ethanol amide (PEA) is an endogenous cannabinoid-like lipid mediator with widely reported anti-inflammatory, analgesic, antimicrobial, immunomodulatory, and neuroprotective effects. It has been demonstrated that PEA corrects mitochondrial dysfunction, restores the hippocampal BDNF signaling pathway, and activates PPAR-α to alleviate autism-like behavior in BTBR T+tf/J mice [109,110].

#### 3.1.3. Clinical Studies

A study investigated 62 patients with ASD using a randomized, double-blind approach. When comparing the RES group to the placebo group, the index of hyperactivity/non-compliance score was much lower, and there was no discernible difference in the quantity and intensity of adverse events between the two groups [75]. Patients with ASD have higher blood concentrations of acylcarnitines given the impairment of mitochondrial fatty acid β-oxidation (mtFAO), the key energy-producing metabolic process that uses fatty acids to create adenosine triphosphate [111]. Baron et al. evaluated how resveratrol and mtFAO affected the fibroblasts of 10 patients with ASD. The findings showed that RES considerably raised mtFAO activity across all research groups [76]. After taking a lignocaine formula—a special combination of lignocaine with quercetin (QUE) and rutin—for 4 months, 37 children with ASD (age range: 4–14 years) showed significant improvements in their health. Approximately 10% more children recovered their speech, 25% more children interacted socially, 50% more children made eye contact and showed attention, and approximately 75% of the participants’ gastrointestinal and allergy symptoms improved, and there were no negative side effects [77]. In a different study, 50 ASD children received a capsule containing 100 mg of lignocaine, 70 mg of QUE, and 30 mg of the QUE glycoside, rutin. The children’s adaptive functioning and general behavior were significantly improved, apart from a brief (1–8 week) increase in irritability [78]. In a recent pediatric case study, the following therapies were administered to a 10-year-old boy with autism in addition to what was prescribed to other children: palmitoyl ethanol amide/lignocaine (co-ultra-PEA-LUT^®^) therapy decreased stereotyping and enhanced clinical performance [79]. Furthermore, another study described the clinical experience of 17 children (three girls and 14 boys), who were treated for degenerative autism with a novel steroid and QUE regimen. The study’s findings demonstrated that supplementing with 250 mg of QUE daily for at least 16 months—that is, one month before stopping steroid treatment—improved social interactions, overall improvement ratings, and certain individuals’ receptive and expressive language abilities [80].

Consequently, it has been demonstrated that a range of polyphenolic substances increase upstream SIRT1 expression and prevent the progression of ASD. Furthermore, polyphenols can show neuroprotective benefits by influencing downstream pathways to enhance mitochondrial biogenesis, suppress inflammation, lower oxidative stress, and limit the development of ASD, the therapeutic mechanism of polyphenols in ASD is shown in Figure 1.

### 3.2. Sleep Disorders

Sleep disorders have been assessed as an emerging global epidemic causing social and economic burden [112]. They are characterized by disruption of circadian rhythms, which seriously affects people’s work and quality of life [113]. Environmentally induced acute and chronic stress stimulates the brain, such as the hypothalamic-pituitary-adrenal (HPA) axis, inducing oxidative stress or neuroinflammation, which further affects the production and release of neurotransmitters, such as 5-hydroxytryptophan, norepinephrine, and dopamine, leading to sleep disorders [114]. This increases the risk of neurodegenerative diseases, cardiovascular diseases, metabolic diseases, and psychiatric disorders [115].

#### 3.2.1. In Vitro Studies

As the most prevalent polyphenols in red, green, and brown algae, phlorotannins (PSs) have been found to possess a variety of biological functions including antioxidant, antidiabetic, anti-aging, anti-inflammatory, anti-allergic, neuroprotective, and memory-enhancement functions [116,117,118]. PSs protect the skin HaCaT cells from PM2.5-induced apoptosis by inhibiting ROS production [53]. One of the most useful and physiologically active components of tea is tea polyphenols (TPs) [119]. Research has shown that TPs prevent apoptosis and shields PC12 cells from methamphetamine (METH) induced loss of cell viability, reactive oxide and nitric oxide generation, and mitochondrial dysfunction. Furthermore, TPs enhance p-ATM and p-Chk2 expression and antioxidant capacity, which in turn can reduce DNA damage and neurotoxicity by triggering DNA repair signaling pathways [55]. *Mentha haplocalyx Briq.*, *Perilla frutescens (L.) Britt.*, rosemary (Rosmarinus officinalis), lemon balm (Melissa officinalis), and other plants contain Rosmarinus acid (RA), a naturally occurring polyphenol that has antiviral, antioxidant, analgesic, neuroprotective, and cardioprotective properties [120,121,122]. It has been shown that the Akt/GSK-3β/Fyn pathway primarily mediates the antioxidant benefits of RA by upregulating Nrf2 activity [57].

#### 3.2.2. In Vivo Studies

A study based on electroencephalography and electromyography sleep analysis showed that PS reversed caffeine-induced sleep disruption in mice well and was superior to the well-known sedative-hypnotic drug zolpidem [54]. The state of the intestinal environment, internal and peripheral circadian rhythm disorders, and cognitive deficits were all positively treated by TPs in a study of mice with sleep disorders brought on by disruptions to the circadian rhythm. This is likely because TPs markedly increased the number of hypothalamic cell clusters, up-regulated the number of astrocytes and fibroblasts, and enhanced the expression of the circadian rhythm genes Cry2, Per3, Bhlhe41, Nr1d1, Nr1d2, Dbp, and Rorb in hypothalamic cells. Simultaneously, TPs corrected structural disruptions in the intestinal flora, which improved homeostatic imbalances and modulated the generation of metabolites linked to tryptophan metabolism, glycolysis/glycolysis, and pyruvate metabolism [56]. Gamma-aminobutyric acid (GABA) plays an important role in regulating sleep. It promotes relaxation and sleep by inhibiting neuronal excitability and reducing neurotransmission and activity [123]. In a recent study, RA (2.0 mg/kg) enhanced total sleep and RA (po) increased protein expression of glutamic acid decarboxylase (GAD65/67) and GABAA receptor subunits other than the β1 subunit in rats, while simultaneously reducing sleep/wake cycles and REM sleep counts. This implies that pentobarbital-induced sleep behavior is enhanced by RA through GABAergic transmission [58].

#### 3.2.3. Clinical Studies

In a one-week, double-blind, randomized study, 24 participants took either PSs (500 mg/day) or a placebo 30–60 min before bed. At baseline and one week later, sleep parameters were measured with polysomnography (PSG) and sleep questionnaires. When compared to placebo, PSs significantly increased “sleep duration” ratings, significantly reduced dyspnea during REM sleep while supine, and had no major side effects in either group [81]. Decaffeinated green tea was shown to lower stress and increase sleep quality in a double-blind crossover design experiment including 20 middle-aged men and women. Standard caffeine was also evaluated for its effects on stress response and sleep metrics [82]. In a different study, 12 non-obese males were divided into three groups and given either 100 mg of caffeine, 100 mg of oolong tea (which contains 100 mg of caffeine, 21.4 mg of gallic acid, 97 mg of catechin, and 125 mg of polymerized polyphenols), or 100 mg placebo over 14 days. On day 14 of every session, PSG was used to assess sleep and indirect calorimetry was used to quantify energy metabolism. The findings demonstrated that while caffeine and oolong tea consumption raised fat oxidation and parasympathetic activity, they had no influence on sleep-related measures [83]. A different randomized controlled parallel experiment shown that in those with subclinical sleep disturbances, RA enhanced sleep quality, reduced insomnia severity, and improved neurocognitive performance compared to placebo [84].

Thus, polyphenols have been shown to improve sleep by improving the gut microflora and enhancing the efficacy of GABAA delivery. In addition, polyphenols may reduce oxidative stress to further prevent neurotoxicity and cognitive impairment due to insomnia. The therapeutic mechanism of polyphenols for sleeping disorders is shown in Figure 2.

### 3.3. Anxiety Disorders

Anxiety disorders are prevalent mental conditions characterized by a fear reaction triggered by potentially hazardous or demanding situations. These illnesses stem from an intricate interaction of biological variables. Anxiety disorders encompass panic disorder/platonic disorder (PDA), generalized anxiety disorder (GAD), social anxiety disorder (SAD), and obsessive compulsive disorder (OCD) [124,125,126]. Anxiety disorders are linked to oxidative stress and changes in the immune system. The most straightforward explanation for the heightened inflammation in post-traumatic stress disorder (PTSD) and GAD is the activation of the stress response and the production of cytokines from immune cells in both the central and peripheral systems [127,128]. Emerging data indicate that pro-inflammatory indicators can directly influence emotional behaviors. By using polyphenols for their antioxidant and anti-inflammatory properties, it is possible to mitigate neuronal damage in the brain and thereby reduce the risk of anxiety disorders.

#### 3.3.1. In Vitro Studies

ROS overwhelm intracellular antioxidant scavenging capacity and lead to oxidative damage to DNA, lipids, and proteins, and subsequently cellular injury [129]. Transcription factor nuclear factor-erythroid 2-like 2 (NRF2) is a major regulator of the antioxidant defense system, modulating the expression of antioxidant proteins and thereby preventing oxidative stress induced by injury and inflammation [130]. Chlorogenic acids (CGA) are a diverse collection of plant polyphenols that possess anti-inflammatory, antioxidant, anticancer, and neuroprotective properties. Research has shown that CGA stimulates Nrf2 and substantially enhances the production of antioxidant proteins such as glutamate cysteine ligase (GCL), heme oxygenase-1 (HO-1), and Sestrin2 [61]. Glutathione (GSH) or reduced glutathione, is a compound made up of three amino acids: gamma-glutamyl-cysteinyl glycine. It is the main antioxidant found inside cells in many creatures, including humans. Transcription factor-nuclear factor-2 related erythroid (Nrf2) regulates the activity of GSH-associated enzymes [131]. *Forsythia suspensa (Thunb.) Vahl* is commonly utilized in traditional medicine to treat a variety of ailments. The administration of HP extract (HpE) effectively inhibited cell death, depletion of GSH, and DNA damage generated by tert-butyl hydroperoxide. This protective effect was achieved by upregulating the levels of Nrf2 in the nucleus [63]. Pre-treatment with curcumin (CUR) can effectively prevent cellular inflammatory injury. It achieves this by inhibiting the production or expression of pro-inflammatory cytokines and proteins such as tumor necrosis factor-α (TNF-α), interleukin 1β (IL-1β), nitric oxide (NO), inducible nitric oxide synthase (iNOS), and cyclooxygenase 2 (COX-2). Additionally, CUR improves cell viability and reduces intracellular levels of CUR, thereby preventing cellular inflammatory damage. Furthermore, CUR significantly mitigates neuronal toxicity by enhancing cell viability, reducing intracellular levels of ROS and malondialdehyde (MDA), and increasing GSH levels [66]. Trans-resveratrol (TRE), a naturally occurring polyphenol found in high concentrations in grape seeds and skins, has been the subject of extensive research because of its potent antioxidant, anti-inflammatory, and antipsychotic characteristics [132]. Studies have shown that TRE can effectively scavenge PM-generated free radicals and mono-linear oxygen 2.5, reduce cell death, and prevent oxidative damage to HaCaT cells, and protect HaCaT cells against the dark and light induced toxicity of PM2.5 [59].

#### 3.3.2. In Vivo Studies

A study conducted on rats with PTSD induced by a time-dependent sensitization (TDS) procedure showed that TRE (the treatment being studied) increases the phosphorylation of cAMP response element binding protein (pCREB) and the levels of BDNF. The study also found that TRE protects neurons from stress-related injuries similar to those seen in PTSD by modulating the function of the L-HPA-axis and activating neuroprotective molecules downstream (such as pCREB and BDNF expression) [60]. In living organisms, free radicals act on lipids to undergo peroxidation, and the oxidation end-product is malondialdehyde, which causes cross-linking and polymerization of proteins, nucleic acids, and other macromolecules, and is cytotoxic. Studies have shown that CGA exerts anxiolytic and neuroprotective effects through inhibition of acetylcholinesterase and malondialdehyde in the hippocampus and frontal cortex and ameliorates scopolamine-induced learning, memory, and cognitive deficits [62]. A study in FG-7142-induced experimental anxiety rats showed that HpE exerts anti-inflammatory (decreased IL-1α, IL-1β, MCP1, IFN, and MIP mainly in the hippocampus) and antioxidant effects (decreased MDA levels and increased CAT and SOD activity), enhances NFκB and pNFκB expression in the brain, and decreases serum corticosterone levels. These results suggest that HpE provides brain protection and ameliorates anxiety-like behaviors by modulating oxidative stress and inflammation [64]. A controlled study in rats exposed to cadmium chloride toxicity-induced anxiety-depression-like rats showed that GSE treatment normalized 5-HTT expression and prevented oxidative damage by increasing glutathione reductase (GR) levels, restoring GST and glutathione peroxidase (GPx) expression, and reducing MDA levels [65]. Research has shown that administering CUR before an event can avoid stress-induced negative changes in behavior. CUR decreases brain MDA concentrations and enhances the activities of CAT, GPx, SOD, and acetylcholinesterase (AChE) [67]. Catechin (-) epigallocatechin-3-gallate (EGCG) is a significant polyphenol that has positive impacts on anxiety and depression. Research has shown that EGCG reduces the negative effects on learning and memory caused by SPS stimulation by preventing the development of neuroinflammation in the rat brain [133]. Protocatechuic acid PCA exerts antidepressant and anxiolytic effects by modulating the serotonergic nervous system and monoamine transmitters in SD rats [134].

#### 3.3.3. Clinical Studies

A total of 79 adults who reported having digestive problems were selected to participate in an 8 week study. The study was designed as a parallel, double-blind, randomized controlled trial. The participants were randomly divided into two groups: one group received a placebo, while the other group received a 500 mg dose of CUR. The results of the study showed that the CUR group experienced significant improvements in their Gastrointestinal Symptom Rating Scale (GSRS) and Depression, Anxiety, and Stress Scale-21 (DASS-21) scores compared to the placebo group [85]. A recent clinical study, utilizing a randomized, double-blind, placebo-controlled design, examined the effects of long-term supplementation with Avena sativa (AS) on cognitive function and physiological responses to stress in 132 healthy individuals. The results demonstrated that AS supplementation led to improved cognitive function and a modulation of physiological responses to stress. Additionally, the AS group exhibited a noteworthy decrease in anxiety symptoms, as measured by the STAI score [86]. Christiane et al. conducted a clinical trial on 78 healthy volunteers using a randomized, double-blind, placebo-controlled study design. They found that consuming GSEe tablets at a dosage of 300 mg per day for 16 weeks resulted in a more significant reduction in experienced stress [87].

Thus, polyphenols can inhibit the onset of neuroinflammation while modulating serotonin and monoamine transmitters in the brain to protect neurons from stress damage to exert anxiolytic and neuroprotective effects, the therapeutic mechanism of polyphenols for anxiety disorders is shown in Figure 3.

### 3.4. Depression

Depression is a widespread mental health condition and “mood disorder” that is defined by emotional symptoms such as sorrow and loss of pleasure, cognitive symptoms such as difficulties with thinking and difficulty with focusing, and physical symptoms such as changes in appetite and sleeplessness. Depression is a psychiatric disorder whose molecular etiology is still unclear. The primary systems presently under investigation encompass monoamines, stress, neurotrophins and neurogenesis, excitatory and inhibitory neurotransmission, mitochondrial dysfunction, genetics, inflammation, opioid system, myelination, and the gut–brain axis.

#### 3.4.1. In Vitro Studies

Cyclic AMP response element-binding protein (CREB) is a transcription factor that is linked to depression. The regulation of multiple protein kinases in the brain directly or indirectly affects the activation of CREB. CREB plays a role in controlling the expression of nerve growth factors and has similar effects to classical antidepressants. It can prevent changes in proteins such as neurotrophic factors and apoptosis in the brain. BDNF is a crucial member of the nerve growth factor family, known for its involvement in cell survival and the ability of neurons to change and adapt. Anthocyanins (ANT) are inherent polyphenolic compounds that bestow fruits, vegetables, and plants with vivid hues and possess diverse neuroprotective characteristics. Anthocyanins reduce the excessive expression of inducible NO synthase, cyclooxygenase-2, TNF-α, and IL-1β in lipopolysaccharide (LPS)-stimulated BV2 cells. Additionally, anthocyanins hinder the movement of nuclear factor-κB (NF-κB) into the nucleus and inhibit Akt by reducing the degradation of inhibitors of NF-κB α and phosphorylating extracellular signal-regulated kinases, among other effects [68]. Ferulic acid (FA) is an abundant phenolic phytochemical found in plants. It possesses anti-apoptotic and antioxidant characteristics, and it mitigates cellular damage caused by H_2_O_2_ by reducing the activation of extracellular signal-regulated kinase (ERK). FA enhances the production of BDNF via modulating the expression of microRNA-10b [72]. The compound 3,5,6,7,8,3′,4′-heptamethoxyflavone (HMF) is a polymethoxyflavone compound that is present in citrus fruits. Research has shown that HMF might potentially enhance neuronal protection by activating the production of m-BDNF and CREB signaling, while also inhibiting the activity of PDE4B or PDE4D [73]. Quercetin regulates the NF-κB/HO pathway to suppress the production of nitric oxide (NO) and inducible nitric oxide synthase (iNOS), hence inhibiting the movement of NF-κB into the cell nucleus and decreasing the expression of heme oxygenase-1 (HO-1). This mechanism helps to prevent cellular inflammation and damage [70].

#### 3.4.2. In Vivo Studies

A study in CUMS-induced mice showed that ANTs significantly improved depressive nurturing behaviors in mice by a mechanism that promotes neurogenic dendritic development by increasing the up-regulation of BDNF through increased mediation of the ERK/CREB/BDNF signaling pathway [69]. BDNF plays a crucial role in promoting neuroprotection by facilitating neuronal plasticity and survival, synaptic transmission, and neurotransmitter production. Studies have shown that RES enhances BDNF levels in several areas of the animal brain, such as the hippocampus. RES administration resulted in elevated levels of pCREB and BDNF in the hippocampus, prefrontal cortex, and amygdala of mice exposed to LPS and rats subjected to CUMS. Additionally, the ERK signaling pathway plays a role in promoting neuronal survival and recovery. Research has demonstrated that abnormalities in ERK signaling cascades are linked to depression, and it has been found that BDNF has antidepressant effects via increasing the activity of ERK pathways [135]. Furthermore, studies have found that RES enhances the activation of pERK in the hippocampus and prefrontal cortex of animals. Hence, RES has the potential to treat symptoms of depression by enhancing the production of BDNF and activating the ERK signaling cascade. Another study showed that QUE effectively reduced the development of behavioral dysfunction in mice subjected to continuous unexpected stress. This was achieved by reducing oxidative and inflammatory stress in the hippocampus. Furthermore, therapy with QUE considerably improved symptoms of depression, eased cognitive dysfunction, and restored normal motor functioning [136]. QUE decreased levels of oxidative stress indicators such as TBARS and nitric oxide, but increased levels of antioxidants such as total thiols and catalase. Additionally, QUE boosted the production of pro-inflammatory cytokines including IL-6, TNF-α, IL-1β, and COX-2 in the hippocampus, leading to damage in hippocampal neurons. The administration of QUE resulted in a considerable decrease in oxidative and inflammatory stress, hence preventing any neurological injury. The HPA axis plays a crucial role in the body’s response to external stress and in maintaining balance in several physiological processes, such as energy metabolism and neuropsychiatric function. Chronic environmental stress can lead to excessive activation of the HPA axis, which in turn can cause neuroinflammation. A study conducted on male rats that were exposed to prenatal stress has shown that FA effectively alleviates depression by inhibiting neuroinflammation through the suppression of IL-6, IL-1β, and TNF-α, as well as by reducing the levels of adrenocorticotropic hormone (ACTH) and corticosterone in the bloodstream [71]. Administering HMF to rats with corticosterone-induced sadness resulted in increased body weight, decreased depression-like behavior, and restored BDNF expression. These findings indicate that HMF functions as an antidepressant via promoting the production of BDNF [74].

#### 3.4.3. Clinical Studies

A recent study examined the consumption of anthocyanins in 93 diverse groups, including those with major depressive disorder (MDD) and healthy individuals. The study found that those with MDD had insufficient intake of anthocyanins in their diet, and increased consumption of anthocyanins was linked to reduced depressive symptoms in the overall group [88]. A recent study conducted on 50 hypertensive patients used a randomized, triple-blind, placebo-controlled crossover design. The participants were evaluated using the Beck Anxiety and Depression Inventory (BAI and BDI). The results showed that those who received either four placebo or celery seed extract capsules (1.34 g/day) for 4 weeks experienced a significant reduction in BAI and BDI scores. The mean reduction in BAI scores was 6.78 (*p* < 0.001), while the mean reduction in BDI scores was 3.63 (*p* < 0.001). These findings indicate that celery seed extract may have a positive effect on anxiety and depression symptoms in hypertensive patients. The study showed that apigenin (AG) effectively alleviated depression symptoms, including melancholy, weeping, dysphoria, sleeplessness, irritability, exhaustion, decreased libido, and thoughts of punishment (*p* < 0.01). Additionally, there was a substantial drop in blood pressure parameters following treatment with AG (*p* < 0.001) [89].

Thus, polyphenols elevate brain neurotrophic factors, alleviate HPA axis hyperactivation, and promote neurogenic dendritic development. In addition, polyphenols can hinder inflammation, decrease oxidative stress, demonstrate neuroprotective properties, regulate subsequent occurrences, safeguard neurons, and avert apoptosis, thereby delaying the progression of depression, the therapeutic mechanism of polyphenols for depression is shown in Figure 4.

## 4. Conclusions and Perspective

There has been a plethora of encouraging evidence from recent investigations about the benefits of polyphenols on nervous system disorders. However, due to the high variability in polyphenols and the large differences in polyphenol content between foods and vegetables, it is difficult to achieve the desired bioactive concentration in the body with food alone. Information on the exact concentration of polyphenols in foods is lacking, and the amount of literature currently available also does not allow for the recommendation of specific forms of medication [137,138]. Therefore, further studies on polyphenols are necessary regarding methods of administration, target tissues, optimal dosage, and optimal composition of phenolic extracts. At the same time, the limited epidemiologic evidence for polyphenols in neurological disorders is acceptable, but a systematic approach, including translational preclinical studies as well as clinical trials using therapeutically relevant dosages, is needed to determine the role of polyphenols in neurological disorders. Polyphenols, due to their natural origin, are generally believed to be non-toxic and safe. Data from clinical and preclinical studies demonstrated that the evaluated phenolic compounds were well tolerated, free of adverse effects and had a high safety profile [139,140]. However, this cannot be established as a rule. More research is needed in order to assess the total toxicity and levels of harmful compounds produced during food processing or polyphenol extraction [141,142].

Polyphenols exhibit substantial neuroprotective properties; nevertheless, their therapeutic use is hindered by a limited comprehension of the underlying operating mechanisms. This review is a comprehensive compilation of the function and processes of polyphenols in the management of nervous system disorders, encompassing both laboratory experiments and investigations involving human subjects. Polyphenols, as multi-targeted medicines, have been discovered to play a vital role in averting the production of harmful substances such as oxidative stress and inflammatory factors. Polyphenols provide two significant benefits as pharmaceuticals for treating nervous system disorders. Polyphenols are naturally generated by plants and can be obtained through regular dietary intake. They have a reduced number of adverse effects and are appropriate for extended periods of usage. Furthermore, polyphenols possess the ability to effectively cure nervous system illnesses by targeting many pathways, a vital aspect in addressing diverse disorders. Nevertheless, polyphenols are infrequently employed as pharmaceutical agents for the management of nervous system disorders. Future research should include extensive multicenter randomized controlled trials and detailed mechanistic investigations to comprehensively assess the safety, effectiveness, and potential adverse effects of polyphenols as medicinal agents.

## Figures and Tables

**Table 1:** Role and mechanisms of polyphenols in the treatment of nervous system disorders.

Compounds	Disease	Model		Effects and Mechanisms		References
		In Vitro	In Vivo	In Vitro	In Vivo	
RES	Autism-spectrum disorder	KGN cells	Mice: VPA	Improvement of mitochondrial quantity through stimulating SIRT1/PGC-1α.	Prevents mPFC neuronal changes, antioxidant and neuroprotective effects, improves E/I balance-related parameters.	[43,44]
		C2C12 cells	Mice: BTBR	Neuroprotection through inducing AMPK activation, regulating SIRT-1 protein activity, and promoting mitochondrial biogenesis.	Restoration of social interaction and enhancement of socialization in mice, improvement of neuroimmune disorders, suppression of molecules that promote inflammation and the signaling pathway involving TLR/NF-κB transcription factors.	[45,46]
BAI		BV-2 cells	Wistar rats: VPA	Improvement of neurocognitive deficits through reversing neuroinflammation, inhibition of HMGB1 release via the SIRT1/HMGB1 pathway, and reducing LPS-induced nuclear translocation of HMGB1.	Enhances postnatal growth and maturity, while also improving motor development, repetitive behaviors, and social impairments in rats who were exposed to VPA during prenatal stages. Improved functionality of mitochondria in neurons, increased sirtuin-1 (SIRT1) levels in brain tissue of VPA rats.	[47,48]
AFE		RAW 264.7 cells	SD rats: VPA	Suppression of inflammation, blocking of LPS-induced NF-κB and MAPK signaling pathways in RAW264.7 macrophages.	Notable enhancements in neurobehavioral alterations seen in the raised plus-T maze, water maze, and rotating rod test, increases the expression of Nrf2 and HO-1, SIRT-1, and LC3, decreases the expression of NFκB.	[49,50]
HES		BV-2 cells	SD rats: sodium fluoride	Antioxidant, anti-inflammatory, and anti-apoptosis, inhibition of the TLR4 /p-NF-κB signaling pathway.	Ameliorates neurobehavioral disorders and protects the nervous system, modulates Nrf2/Tlr4/NFκB signaling.	[51,52]
PSs	Sleep disorder	HaCaT cells	Mice: caffeine	ROS inhibition, anti-oxidative stress, inhibition of the MAPK signaling pathway.	Relief of transient insomnia symptoms, promoting sleep by regulating GABA.	[53,54]
TPs		PC12 cells	Mice: inversion light/dark cycle	Anti-oxidative stress, increases p-ATM and p-Chk2 expression, activates DNA repair signaling pathway.	Enhancement of internal and peripheral circadian rhythm abnormalities and cognitive impairment, enhances the quantity of hypothalamic cell clusters, increases the expression of astrocytes and fibroblasts, and ameliorates structural abnormalities in the intestinal microbiota.	[55,56]
RA		PC12 cells	SD rats: pentobarbital	Antioxidant stress, mediates Akt/GSK-3β/Fyn pathway activation of Nrf2.	Decreased sleep/wake cycle and REM sleep counts, increased sleep duration, increased glutamic acid decarboxylase and GABAA receptor expression.	[57,58]
TRE	Anxiety disorder	HaCaT cells	SD rats: TDS	Reduces cytotoxicity and reduces apoptosis, protects against oxidative stress.	The TDS-induced decreases in the proportion of time spent in the middle of the arena, open-arm entrance, and time spent in the arena with open arms in the open field and raised cross maze tests were reversed. Reverses the index of adrenal activity and levels of corticotropin-releasing factor (CRF), while enhancing the phosphorylation of cyclic AMP response element-binding protein (pCREB) and levels of brain-derived neurotrophic factor (BDNF).	[59,60]
CGA		HepG2 cells	Mice: SCOP	Antioxidant stress, activation of Nrf2, *ARE* gene, and GCL, HO-1 and Sestrin2 expression.	Enhances short-term or working memory impairments in the scopolamine-induced Y-maze test, effectively counteracts cognitive impairments in the passive avoidance test in mice, and decreases the time taken to escape in the Morris water maze test, enhances GABA activity, and avoids neurological harm.	[61,62]
HpE		HepG2 cells	Wistar rats: FG-7142	Cryoprotection, activation of Nrf2 and increases GSH levels.	Improves anxiety behavior, modulates oxidative stress and inflammatory response, reduces IL-1α, IL-1β, MCP1, IFN, and MIP, reduces MDA levels, increases CAT and SOD activity, reduces CORT levels.	[63,64]
GSE			SD rats: Cd		Increases glutathione reductase (GR) levels, restores GST and GPx expression, and decreases MDA levels to prevent oxidative damage. Restores 5-HTT expression.	[65]
CUR		SH-SY5Y cells	Wistar rats: immobilization stress	Inhibition of cellular inflammatory damage. Increased PPARγ protein expression, increased activity of ROS scavenging enzymes SOD and CAT.	Improves anxiety behavior, prevents stress-induced behavioral deficits, improves memory, reduces brain MDA levels, elevated CAT, GPx, SOD, and AChE activities.	[66,67]
ANT	Depression	BV2 microglial cells	Mice: CUMS	Anti-inflammatory effect, blocking activation of NF-κB, PI3K/Akt, and MAPK signaling cascade responses in microglia.	Depression-like behavior was significantly improved after CUMS treatment. Mediation of the ERK/CREB/BDNF signaling pathway was enhanced, which upregulated BDNF and promoted neuronal dendrite development.	[68,69]
QUE		SH-SY5Y cells	Mice: CUS	Modulation of the NF-κB/HO pathway to inhibit NO and iNOS expression. Prevents NF-κB nuclear translocation and HO-1 downregulation.	Markedly decreased anxiety, relieved sadness, improved cognitive impairment, and restored normal motor functioning. Decreased concentrations of indicators of oxidative stress. The levels of TBARS, NO, and antioxidants (total thiols, catalase) were increased. Decreased production of pro-inflammatory cytokines (IL-6, TNF-α, IL-1β, and COX-2) in the hippocampus and injured hippocampal neurons.	[70,71]
FA		PC12 cells	SD rats: PD	Resistance to oxidative stress in PC12 cells. Inhibits phosphorylation of ERK to attenuate H_2_O_2_-induced cellular damage. Increases BDNF by regulating microRNA-10b expression.	Amelioration of depressive-like behavior in rats descended from prenatal stress, inhibition of IL-6, IL-1β, and TNF-α, increase in IL-10 mRNA and protein expression, and significant reduction in adrenocorticotropic hormone (ATH) and adrenocorticotropic hormone (ATH) concentrations.	[71,72]
HMF		C6 cells	Mice: CUMS	Neuroprotective effect, induction of m-BDNF expression to exert its neuroprotective effect/CREB signaling. Inhibits PDE4B or PDE4D.	Amelioration of corticosterone-induced weight loss and depressive-like behavior, up-regulation of BDNF in the hippocampus via the ERK1/2/MAP system.	[73,74]

RES, resveratrol; BAI, baicalin; AFE, aqueous fruit extract; HES, hesperidin; PSs, phlorotannins; TPs, tea polyphenols; RA, Rosmarinus acid; TRE, trans-resveratrol; CGA, chlorogenic acids; HpE, hypericum perforatum extract; GSE, grape seed extract; CUR, curcumin; ANT, anthocyanins; QUE, quercetin; FA, ferulic acid; HMF, heptamethoxyflavone.

**Table 2:** A clinical study of the use of polyphenols in the treatment of nervous system disorders.

Compounds	Disease	Type of Study	Sample Size	Treatment Schedule	Finding	Reference
RES	Autism-spectrum disorder	Randomized double-blind controlled trials	62	250 mg RES twice daily for 10 weeks	No significant impact on irritation, improved ASD hyperactivity/noncompliance.	[75]
		Randomized trial ex vivo study	10	RES 2 mg kg-1 per day, up to 50 mg per day; 12 weeks total treatment	Resveratrol substantially boosted mtFAO activity, particularly in fibroblasts from individuals with severe symptoms.	[76]
Luteolin		Case series of study	37	A total of 200 mg of lutein once daily for at least 4 months	Improved speech recovery by 10%, social interaction by 25%, eye contact and attention by 50%, and gastrointestinal and allergy problems in 75% of subjects without side effects.	[77]
		Prospective open label trial	50	200 mg Luteolin per 10 kg body weight for 26 weeks	Significantly enhanced children’s adaptive functioning and conduct, transient (1–8 weeks) irritation, no serious adverse effects.	[78]
		Case report	1	Co-ultraPEA-LUT^®^ at a dose of 700 mg + 70 mg	Improved clinical picture and stereotype reduction in a 10-year-old boy.	[79]
QUE		Not mentioned	17	Supplementation with 250 mg of quercetin per day for 16 months	Some autistic individuals improved their global progress score, social interaction, receptive language, and expressive language.	[80]
PSs	Sleep disorder	Randomized double-blind placebo-controlled trial	24	500 mg/day for 1 week	PSs significantly increased “sleep duration” ratings and decreased dyspnea during supine REM sleep without major side effects.	[81]
TPs		Double-blind crossover design	20	Tea consumption (≥300 mL/day) within 7 days	Reduces stress, improves sleep quality.	[82]
		Randomized, placebo-controlled, double-blind crossover trial	12	Oolong tea (100 mg caffeine, 21.4 mg gallic acid, 97 mg catechins, 125 mg polymerized polyphenols) was eaten for 14 days	Increased fat oxidation but no improvement in sleep.	[83]
RA		Randomized controlled parallel trials.	89	65 mg daily for 30 days	EGCG and RA enhanced sleep and decreased insomnia.	[84]
CUR	Anxiety disorder	Parallel double-blind randomized controlled trials	77	500 mg CUR extract for 8 weeks	Notable enhancement in the Gastrointestinal Symptom Rating Scale (GSRS) and the Depression, Anxiety and Stress Scale-21 (DASS-21)	[85]
AS		Randomized double-blind placebo-controlled trial	132	430 mg, 860 mg and 1290 mg over 29 days	Long-term supplementation may benefit cognitive function and modulate physiological responses to stressors. Significant reduction in observed anxiety symptoms using the STAI score.	[86]
GSE		Randomized double-blind, placebo-controlled trials	78	300 mg/day for 16 weeks	GSE relieved perceived stress.	[87]
ANT	Depression	not mentioned	93	Different anthocyanin intake	Dietary deficiencies in ANT may cause major depression.	[88]
AG		Randomized triple-blind placebo-controlled crossover trial	50	1.34 g/day for 4 weeks	Improves BAI and BDI and reduces depression symptoms such as sorrow, weeping, trance, sleeplessness, irritability, exhaustion, loss of libido, and thoughts of punishment.	[89]

AS, Avena sativa; AG, Apigenine.

## Data Availability

Data are contained within the article.
